# Clinical utility of convolutional neural networks for treatment planning in radiotherapy for spinal metastases

**DOI:** 10.1016/j.phro.2022.02.003

**Published:** 2022-02-17

**Authors:** Sebastiaan R.S. Arends, Mark H.F. Savenije, Wietse S.C. Eppinga, Joanne M. van der Velden, Cornelis A.T. van den Berg, Joost J.C. Verhoeff

**Affiliations:** a Department of Radiotherapy, Division of Imaging & Oncology, University Medical Center Utrecht, Utrecht, The Netherlands; b Computational Imaging Group for MR Diagnostics & Therapy, Center for Image Sciences, University Medical Center Utrecht, Utrecht, The Netherlands

**Keywords:** Spinal metastases, Deep learning, Artificial intelligence, Auto-segmentation

## Abstract

**Background and purpose:**

Spine delineation is essential for high quality radiotherapy treatment planning of spinal metastases. However, manual delineation is time-consuming and prone to interobserver variability. Automatic spine delineation, especially using deep learning, has shown promising results in healthy subjects. We aimed to evaluate the clinical utility of deep learning-based vertebral body delineations for radiotherapy planning purposes.

**Materials and methods:**

A multi-scale convolutional neural network (CNN) was used for automatic segmentation and labeling. Two approaches were tested: the combined approach using one CNN for both segmentation and labeling, and the sequential approach using separate CNN’s for these tasks. Training and internal validation data included 580 vertebrae, external validation data included 202 vertebrae. For quantitative assessment, Dice similarity coefficient (DSC) and Hausdorff distance (HD) were used. Axial slices from external images were presented to radiation oncologists for subjective evaluation.

**Results:**

Both approaches performed comparably during the internal validation (DSC: 96.7%, HD: 3.6 mm), but the sequential approach proved more robust during the external validation (DSC: 94.5% vs 94.4%, p < 0.001, HD: 4.5 vs 7.1 mm, p < 0.001). Subsequently, subjective evaluation of this sequential approach showed that experienced radiation oncologists could distinguish automatic from human-made contours in 63% of cases. They rated automatic contours clinically acceptable in 77% of cases, compared to 88% of human-made contours.

**Conclusion:**

We present a feasible approach for automatic vertebral body delineation using two variants of a multi-scale CNN. This approach generates high quality automatic delineations, which can save time in a clinical radiotherapy workflow.

## Highlights

• We presented a CNN workflow for segmentation and labeling of vertebrae on CT.
• This approach proved to be robust in a majority of cases with spinal metastases.
• The presented workflow can save time in a clinical radiotherapy setting.
• The approach also allows for more advanced quantitative image analysis of vertebrae.

## 1 Introduction

Spinal metastases are common in patients with cancer and can have serious quality of life limiting consequences including pain, pathologic fractures and spinal cord compression [1]. Radiotherapy, including stereotactic body radiotherapy (SBRT), successfully reduces pain in the majority of patients [2].

Since the introduction of high conformal treatments like Intensity Modulated Radiotherapy (IMRT) and Volumetric-Modulated Arc Therapy (VMAT), an essential step in radiotherapy treatment planning is structure delineation on medical images, usually computed tomography (CT). Structure delineations are needed to optimize planned radiation dose in the tumor and minimize radiation dose in organs at risk. Consequently, clinical outcome of radiotherapy is dependent on the quality of structure delineations.

Manual delineation of structures is time-consuming and susceptible to interobserver variability [3]. Unsurprisingly, automatic structure delineation has received great scientific interest [4]. Automatic spine delineation can be divided into two separate tasks: segmentation (correctly distinguishing vertebrae from background) and labeling (correctly identifying each vertebral level). Various approaches for automatic spine delineation on CT have been proposed, yielding promising results [5], [6], [7], [8], [9], [10], [11], [12], [13].

In recent years, deep learning has increasingly become the methodology of choice for automatic structure delineation due to its favorable performance: reliable and fast output [14]. Deep learning is a form of machine learning and uses neural networks with multiple layers to progressively extract higher level features from raw input [15]. More specifically, in medical image analysis, convolutional neural networks (CNN) are mainly used [14].

Although automatic spine delineation using deep learning yielded promising results in publicly available datasets from healthy subjects [10], the utility for clinical radiotherapy practice is unclear. The objective of this study was to investigate the clinical utility by quantitatively and subjectively evaluating the quality of CNN-generated vertebral body delineations for radiotherapy planning purposes. To achieve this, we trained and validated multi-scale CNN’s using images directly from clinical practice.

## 2 Materials and methods

### 2.1 Training and internal validation data

The CT image series used to develop the automatic delineation method were selected from the PRESENT cohort that includes all patients with bone metastases referred to the radiation oncology department in the University Medical Center Utrecht [16]. From this cohort, a random selection of 60 scans was made using the following criteria: presence of bone metastases in the trunk (vertebrae, ribs, sternum and/or pelvis); CT slice thickness of 1 mm; visibility of at least 5 thoracic and/or lumbar vertebrae; absence of artefacts (e.g. due to surgically inserted metal). The 60 scans were divided into four groups of 15, to perform four-fold cross-validation, with each fold containing 45 training and 15 test scans.

Altogether, the selection included scans from 59 unique patients (31 female, 28 male). One male patient was included twice in the selection, but with sequential treatments and different regions of the spine visualized on each CT scan. The dataset comprised 639 thoracolumbar vertebrae. Of these vertebrae, 580 were fully visualized, whereas the other 59 were only partially visible at the edges of the scans. The number of vertebrae per vertebral level ranged from 28 to 42, exact numbers are displayed in Supplementary Table 1. Because in our institute CT scans with 1 mm slice thickness are only used in treatment planning for SBRT, all selected patients were treated using this high-precision radiotherapy technique. The study protocol for PRESENT was approved by the Institutional review and Ethics board of the University Medical Center Utrecht (approval number 13–261/D).

All images were acquired between November 2014 and December 2019 using a Brilliance Big Bore CT (Philips, Best, the Netherlands), which has a reconstruction matrix of 512 × 512 voxels. Size of reconstructed voxels was equal in anterior-posterior and right-left directions in the range 0.78–1.37 mm, depending on the used field-of-view. As a result of the selection criteria, slice thickness was 1 mm.

Vertebrae were manually delineated by a single observer (SA) using RayStation v8.99.30.40 (RaySearch Laboratories AB, Sweden). All thoracic and lumbar vertebrae were delineated, in which 80–90% of spinal metastases occur [17], [18]. To limit the required time, the vertebral body was delineated, as this is the predominant location of spinal metastases [19].

### 2.2 External validation data

After the four folds of internal validation, the networks were retrained on all 60 scans and evaluated using the publicly available VerSe 2019 dataset [20]. This dataset contains 160 CT image series with spine delineations. At the time of external validation, image series and delineations were available only for the 80 training scans. From these scans, a selection was made using the following criteria: visibility of at least 5 thoracic and/or lumbar vertebrae; absence of artefacts (e.g. due to surgically inserted metal); image quality comparable to training data (subjective assessment). Of the resulting 32 scans, 15 were chosen randomly to form the external validation set. These scans included 202 fully visualized thoracolumbar vertebrae, with 8 to 15 vertebrae per vertebral level. Slice thickness of these image series was 0.9–1.0 mm, while sagittal resolution varied between 1 and 3 mm. Because the delineations included the entire vertebrae instead of just the vertebral body, the vertebral arch was manually removed from the segmentations.

### 2.3 Network architecture and training

**Fig. 1:**
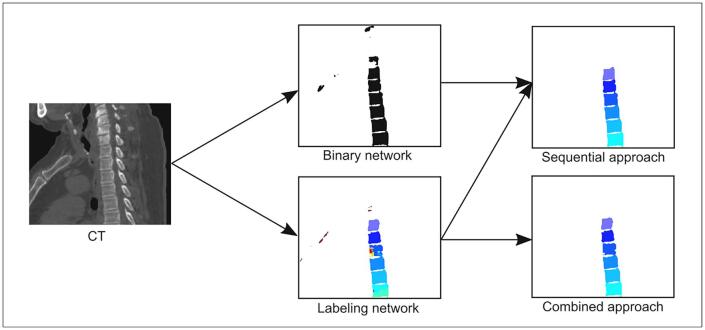
Overview of networks and approaches. Different colors represent different vertebral labels. Left: sagittal CT image of the thoracic spine. Center: black and white projection of output from the binary network and color output from the labeling network where every vertebra has a distinct color. Right top: sequential approach output from binary and labeling networks; and right bottom: combined approach output.

Two variants of a CNN were trained, one performing both segmentation and labeling (identification), another only performing segmentation. These networks will respectively be referred to as *labeling network* and *binary network*. The inferences by these networks were subsequently used to study the performance of two different approaches for automatic spine delineation. The *sequential approach* uses the binary network for segmentation and sequentially the labeling network for labeling of the vertebrae. The *combined approach* uses the labeling network for both segmentation and labeling. Although the sequential approach is computationally more expensive, it was expected to yield better performance. An overview of the networks and approaches is depicted in Fig. 1.

For creation and training of the networks, DeepMedic [21] was used. DeepMedic^1^ is software for a three-dimensional CNN with patch-based training, originally designed for brain lesion segmentation. To combine local and larger contextual information, a parallel pathway operating on down-sampled image patches is employed. For a more detailed description we refer to the original article by Kamnitsas et al. [21].

Architecture was the same for both the labeling and binary networks, except for the number of output classes: two for the binary network, eighteen for the labeling network. In addition to the original pathways with receptive fields of 17^3^ and 51^3^ voxels, a third, even lower resolution pathway was added with a receptive field of 85^3^ voxels. This way, image patches with varying resolutions are combined to incorporate information from different scales. The largest receptive field corresponds to a cube of 8.5 cm, when centered at a vertebral body this would include (parts of) adjacent vertebral bodies and surrounding tissues such as lung. Training configuration, as shown in Supplementary Table 2, was identical for both networks and largely the same as in a previous study of Savenije et al. [22]. Dice coefficient was used as loss function. All images and delineations were resampled to a voxel size of 1 × 1 × 1 mm and normalized to a range of −0.5 to 1.5 before being supplied to the network.

### 2.4 Post-processing

Inferences by both networks were post-processed to obtain the final delineations. Post-processing consisted of improving the segmentation and labeling of the vertebrae.

For the sequential approach, post-processing started with the output of the binary network. Potential vertebrae were detected using the sizes and positions of the segmented regions and distances between the regions. Because sometimes vertebrae were connected in the segmentation, a watershed algorithm [23] was used to separate any connected vertebrae.

The improved binary segmentation was then combined with the labeled segmentation to create the final labeled segmentation. The labeled segmentation was used to determine the most likely label in each of the vertebral regions of the binary segmentation. The most confident label predictions (95% or more of the vertebra has the same label) were considered to be true, and the other vertebrae were labeled accordingly.

For the combined approach, the labeled segmentation was first converted to a binary segmentation and improved as described above. This improved binary segmentation was then combined with the original labeled segmentation to create the final labeled segmentation. A detailed description of post-processing is included in the Supplementary Material.

### 2.5 Quantitative evaluation

Segmentation performance was evaluated on the internal and external validation data in terms of Dice similarity coefficient (DSC) and Hausdorff distance (HD). DSC and HD were calculated for each vertebra separately, both for correct and incorrect labels. Labeling performance was measured as proportion of correctly labeled vertebrae. In case of incorrectly labeled vertebrae, segmentation performance was measured as if they were correctly labeled when possible. For example, if two vertebrae are not adequately separated in the binary segmentation, both vertebrae will receive the same label. As a result of post-processing, all vertebrae above or below it will be offset by one from the correct label. In this case, for all vertebrae excluding the ones causing the incorrect labels, DSC and HD were calculated as if the labels were correct.

### 2.6 Subjective evaluation

Based on the method proposed by Gooding et al. [24], a subjective evaluation was designed. This evaluation was performed for the approach showing the most accurate results on the quantitative evaluation. For each vertebral level from the first thoracic vertebra to the fifth lumbar vertebra, six slices were randomly selected from the external validation scans, three containing a human segmentation, three containing an automatic segmentation. These images were presented in a random order to two experienced radiation oncologists. They were asked whether the contours were drawn by a human or computer, and how they would rate the contours: (1) large, obvious errors; (2) minor errors that need to be corrected for high precision radiotherapy; (3) minor, clinically not significant errors; (4) precise.

The rationale behind this approach is that quantitative measures such as DSC and HD are not sufficiently capable of distinguishing systematic from random errors [25]. These measures compare the automatic delineations to some “ground truth”, even though this ground truth is subject to inter- and intraobserver variability. The subjective assessment we performed, did not focus on how similar contours are to the ground truth, but on what proportion is deemed clinically acceptable compared to the ground truth, thus minimizing the effect of inter- and intraobserver variability. As shown by Gooding et al. [24], this type of assessment has a stronger correlation with time saved by automatic delineations than quantitative measures.

### 2.7 Statistical analysis

Statistical analysis was performed using R (version 4.0.2). Wilcoxon signed ranks tests were performed for comparison of DSC and HD values. Chi-squared tests were performed for analysis of the subjective assessment. A p-value of 0.05 or less was considered statistically significant.

## 3 Results

**Fig. 2:**
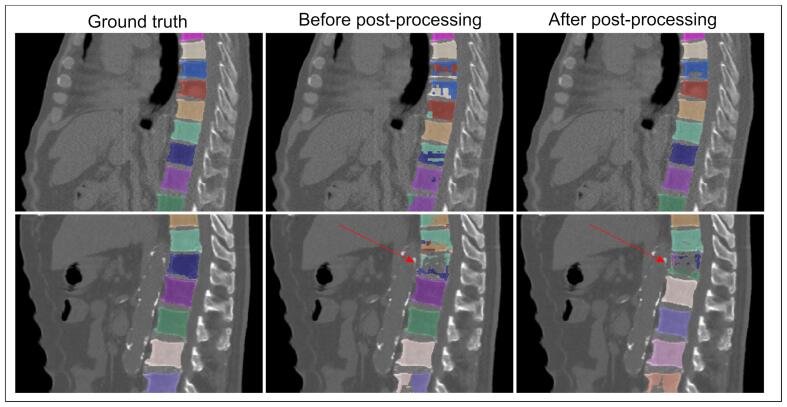
Examples of automatic delineations (using the combined approach) before and after post-processing, projected on the corresponding sagittal CT image in bone setting (W = 2500, L = 1000). Different colors represent different labels. Top row: mixed and incorrect labels in a part of the spine are corrected by post-processing. Bottom row: due to incorrect segmentation of one vertebra (arrows), all vertebrae below are labeled incorrectly even though they were largely correct before.

Computation time was less than 5 min per network. In general, post-processing improved the delineations, but in 10–15% of cases it resulted in incorrectly changed labels because of segmentation errors. This is illustrated in Fig. 2.

**Fig. 3:**
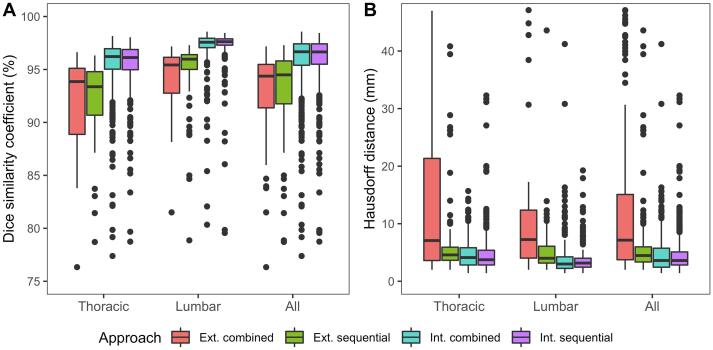
Quantitative assessment: boxplots of DSC (A) and HD (B) values for both the internal (int.) and external (ext.) validation. Both approaches performed similarly during the internal validation, but the sequential approach outperformed the combined approach during the external validation.

Quantitative assessment was performed only for the 580 plus 202 fully visible vertebrae. The results of this evaluation are summarized in Table 1 and Fig. 3. No difference in segmentation performance was seen during the internal validation, but the sequential approach outperformed the combined approach during the external validation (DSC: 94.5% vs 94.4%, p < 0.001, HD: 4.5 vs 7.1 mm, p < 0.001). In general, better segmentation performance was seen for lumbar vertebrae (DSC difference: between +1.4% and +2.6%, HD difference: between +0.1 and –1.1, compared to thoracic vertebrae). More detailed segmentation results per vertebral level are available in Supplementary Tables 3 and 4 for the internal and external validation respectively. Labeling performance was comparable during the internal validation, but a significant drop in performance was observed during the external validation for both approaches (sequential approach: 90.7% vs 79.6%, combined approach: 91.6% vs 55.7%).

**Table 1:** Quantitative assessment: median (inter-quartile range) Dice similarity coefficient (DSC), Hausdorff distance (HD) and proportion (95% CI) of correctly labeled vertebrae. The sequential approach outperformed the combined approach during the external validation.

	**Sequential approach**	**Combined approach**	**p**
**Internal validation**			
**DSC (%)**			
Thoracic vertebrae	96.1 (95.0–96.9)	96.2 (95.0–97.0)	0.003
Lumbar vertebrae	97.6 (97.3–97.9)	97.6 (97.1–97.9)	0.004
All vertebrae	96.7 (95.5–97.4)	96.7 (95.4–97.4)	0.13
**HD (mm)**			
Thoracic vertebrae	3.7 (2.8–5.4)	4.1 (2.8–5.8)	0.45
Lumbar vertebrae	3.2 (2.4–4.0)	3.0 (2.2–4.2)	0.72
All vertebrae	3.6 (2.8–5.1)	3.6 (2.4–5.7)	0.66
**Labeling (%)**			
All vertebrae	90.7 (88.3–93.1)	91.6 (89.3–93.8)	

**External validation**			
**DSC (%)**			
Thoracic vertebrae	93.4 (90.7–94.8)	93.9 (88.9–95.1)	0.09
Lumbar vertebrae	96.0 (95.0–96.4)	95.4 (92.8–96.1)	<0.001
All vertebrae	94.5 (91.8–95.8)	94.4 (91.4–95.5)	<0.001
**HD (mm)**			
Thoracic vertebrae	4.6 (3.6–5.9)	7.1 (3.6–21.3)	<0.001
Lumbar vertebrae	4.0 (3.2–6.1)	7.2 (4.0–12.4)	<0.001
All vertebrae	4.5 (3.4–6.0)	7.1 (3.7–15.1)	<0.001
**Labeling (%)**			
All vertebrae	79.6 (74.0–85.2)	55.7 (48.9–62.6)

**Fig. 4:**
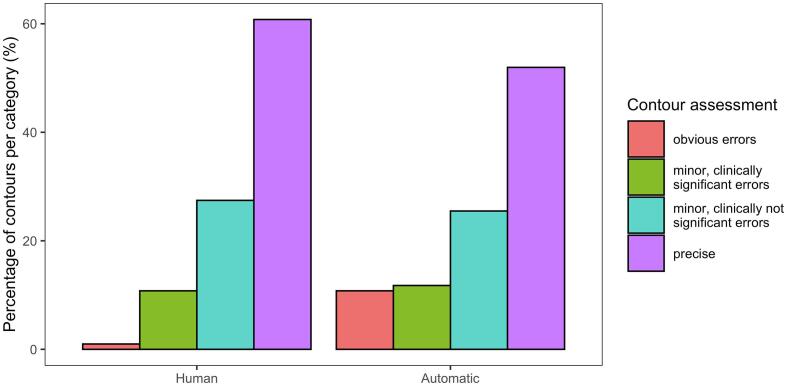
Subjective assessment of contours by radiation oncologists. DL contours were more often rated as having obvious errors (1% vs. 11%, p = 0.004), whereas human-made contours were more frequently considered precise (61% vs. 52%, p = 0.40). In total, 88% of human-made contours were deemed clinically acceptable, compared to 77% of automatic contours.

For subjective assessment, the sequential approach was used. The radiation oncologists correctly determined whether contours were made by a human or automatically in 63% of cases. As Fig. 4 shows, the human-made contours were in general rated to be of higher quality than the automatic contours (p = 0.03). Minor error rates (both clinically significant and not significant) were comparable for both sets of contours. Automatic contours were more often rated as having obvious errors (1% vs. 11%, p = 0.004), whereas human-made contours were more frequently considered precise (61% vs. 52%, p = 0.40). 88% of human-made contours were deemed clinically acceptable, compared to 77% of automatic contours.

## 4 Discussion

Two approaches to automatically delineate thoracolumbar vertebral bodies using CNN’s were implemented and compared for clinical use in a spinal radiotherapy treatment workflow. The sequential approach, using one network for segmentation and another for labeling, demonstrated to be more robust when assessed quantitatively. Subsequently, subjective assessment of this sequential approach showed that automatic delineations were difficult to distinguish from human-made contours by experienced observers. The automatic contours were rated as acceptable even for high precision radiotherapy in 77% of cases, compared to 88% of human-made contours. Since images directly from clinical practice were used, these results suggest that clinical implementation of this technique will lead to a significant reduction in the time needed to delineate vertebral bodies. In most cases, especially for palliative treatments, the contours can be used directly, without further editing. In many other cases, automatic contours are also expected to save time, since they provide a reasonable base upon which improvements can be made.

Previous studies on automatic spine delineation on CT images [5], [6], [7], [8], [9], [10], [11], [12], [13] reported DSC values in the range of 89–96% and HD in the range 5.8–15 mm, although HD was not always reported. Our sequential approach resulted in DSC values of 97% and 95%, and HD values of 3.6 and 4.5 mm for the internal and external validation respectively. Therefore, the proposed method performs better than or comparable to previous studies. Lessmann et al. reported 93% accuracy of labeling on CT images [10]. The sequential approach achieved 91% and 80% accuracy on the internal and external validation respectively.

The strength of this study lies in the focus on application of automatic spine delineation for radiotherapy treatment planning. Previous studies were conducted mainly on small sets of healthy subjects [5], [6], [7], [8], [9], [10], [11], [12], [13]. However, clinical application of automatic delineation would require the technique to be accurate also for patients with pathological vertebrae, such as spinal metastases and fractures. To achieve this, we used images directly from clinical practice for training and evaluation of the network. Moreover, most previous studies only used quantitative measures to evaluate the performance of their approaches, while the relationship between these measures and clinical utility in radiotherapy practice is limited [24]. We chose to evaluate our approach using subjective assessment of delineations in addition to quantitative measures, as this is a better surrogate measure of clinical utility.

Several limitations of this study must be discussed to allow accurate interpretation of the results. The most important concern is that segmentation results could have been positively influenced by only delineating the vertebral body. Although segmentation of the vertebral arch is not necessarily needed for many radiotherapy treatment plans, most other approaches of automatic spine delineation did include it. For example, Yao et al. [13] reported DSC of 94.7%, 96.4% and 91.7% for whole vertebra, vertebral body and vertebral arch segmentation respectively. In addition, only thoracic and lumbar vertebrae were delineated, which could have had a positive impact on the results as well. Hanaoka et al. [7] reported inferior segmentation results for cervical vertebrae and equal results for the sacrum when compared to thoracic and lumbar vertebrae. Nevertheless, most previous attempts of automatic spine delineation were also limited to segmentation of (thoracic and) lumbar vertebrae. Also, including the DSC and HD values for some incorrectly labeled vertebrae as if they had been labeled correctly, might have biased the results. Another limitation is that the external validation set did not include patients with vertebral metastases. This might have led to an overestimation of the results. Furthermore, no hyperparameter search was performed. Hyperparameter tuning could have led to improved segmentation performance. Similarly, post-processing can probably be improved, because it caused large errors (mostly related to labeling) in some cases. A different approach, for example one where segmentation errors are detected and corrected might improve performance. Finally, we chose to only use image series with a slice thickness of 1 mm or less to ensure that vertebrae could be distinguished from one another. Further research is needed to study the impact of 2 or 3 mm slice thickness (commonly used in clinical practice) on the results of our approach.

This study has shown that CNN’s can be used to generate high quality automatic delineations of thoracolumbar vertebral bodies, which are often indistinguishable from human-made delineations. Since radiotherapy treatment planning always occurs under human supervision, complete automation is not required. Despite the potential for even further improvement, the sequential approach presented here is already likely to save precious time if implemented in a clinical workflow. Ideally, the method should delineate not only thoracic and lumbar vertebrae, but also cervical vertebrae and the sacrum (and perhaps even more bone metastasis-prone structures, such as the pelvis, ribs and sternum). Likewise, delineation of full vertebrae is preferred over delineation of vertebral bodies only.

Although the focus of this study was on using automatic delineations for radiotherapy treatment planning, many other applications are conceivable as well. An example related to radiotherapy is to automatically calculate the spinal instability neoplastic score (SINS) and thereby reduce the workload of clinicians and enable better treatment selection [26]. Other examples are automatic osteoporosis detection [27] and spine surgery planning [28]. For some applications, error margins are larger than for radiotherapy treatment planning, and automatic delineations might even be used without manual corrections. Adapted evaluation is needed to determine the accuracy for these applications.

In conclusion, we present a feasible approach for automatic vertebral body delineation using two variants of a multi-scale CNN. This approach generates high quality automatic delineations, which can be useful in a clinical radiotherapy workflow.

## Declaration of Competing Interest

The authors declare that they have no known competing financial interests or personal relationships that could have appeared to influence the work reported in this paper.

## Footnotes

1 Available at https://github.com/Kamnitsask/deepmedic.
