# Supplementary material for: Clinical utility of convolutional neural networks for treatment planning in radiotherapy for spinal metastases
Source: Phys Imaging Radiat Oncol. 2022 Feb 17;21:42–7. doi: 10.1016/j.phro.2022.02.003 (PMC8857663; doi:10.1016/j.phro.2022.02.003)
Supplement: Supplementary Data 1 [file mmc1.docx]

**Clinical utility of convolutional neural networks for spinal radiotherapy treatment planning**

***Supplementary material***

**Table S1 |** Number of fully visible vertebrae per vertebral level.

| **Vertebral level** | **Internal data** | **External data** |
| --- | --- | --- |
| **T1** | 31 | 8 |
| **T2** | 32 | 9 |
| **T3** | 32 | 10 |
| **T4** | 33 | 10 |
| **T5** | 31 | 10 |
| **T6** | 31 | 10 |
| **T7** | 28 | 10 |
| **T8** | 29 | 10 |
| **T9** | 34 | 13 |
| **T10** | 38 | 14 |
| **T11** | 41 | 14 |
| **T12** | 41 | 15 |
| **L1** | 42 | 14 |
| **L2** | 38 | 14 |
| **L3** | 37 | 14 |
| **L4** | 32 | 14 |
| **L5** | 30 | 13 |

**Table S2 |** Training configuration

| **Hyperparameter** | **Configuration** |
| --- | --- |
| Epochs | 35 |
| Batch size | 10 |
| Learning rate | 10^-3^ |
| Optimizer | RMSprop ($\rho=0.9, \varepsilon={10}^{-4}$) |
| Momentum | 0.6 |
| $\mathcal{L}_{1}$regularization | 10^-6^ |
| $\mathcal{L}_{2}$regularization | 10^-4^ |
| Loss function | Dice coefficient |
| Reflection in sagittal plane | 50% |
| Image intensity shift | Gaussian ($\mu=1, \sigma=0.01$) |

**Detailed description of post-processing**

Inferences by both networks were post-processed to obtain the final predicted delineation. Post-processing consisted of (1) improving the initial binary segmentation and (2) labeling the vertebrae.

For the sequential approach, combining the networks, post-processing started with the initial segmentation from the binary network. Within this binary segmentation, small holes were filled and all connected regions were detected. As the size of most vertebrae used in this study was in the range 7-23 cm^3^, all regions larger than 5 cm^3^ were then selected as potential vertebrae. The anterior-posterior (AP) and left-right (LR) coordinates of the centers of these regions were then compared to each other to detect non-vertebra regions. The medians of these values were used as a reference. Regions of which the center is >1.5 cm in LR direction or >4.5 cm in AP direction away from the middle region were considered to be an incorrect segmentation and were removed. These distances were chosen based on the observed distances of true vertebrae.

Subsequently, all remaining regions were inspected to detect potential partly visible vertebrae, as these can be smaller than 5 cm^3^. In order to do this, bounding boxes were determined for both the inspected region and the previously identified vertebrae. A bounding box is the smallest possible box in which the region(s) fit(s). A region was considered to be a partially visible vertebra if its size is >50 mm^3^ and if its distance from the previously identified vertebrae is <0.5 cm in LR direction, <1 cm in AP direction and <0.5 cm in superior-inferior (SI) direction, from either the previously identified vertebrae or the edge of the scan.

As a final check, all identified vertebrae were once again checked in all directions to remove any non-vertebra regions. As before, the LR and AP coordinates of all centers were compared to the medians with 1.5 and 4.5 cm as boundaries respectively. For checking the SI direction, the bounding boxes of all potential vertebrae were used. Starting with the middle region, a region was considered to be a true vertebra if its highest point is within 1 cm of the lowest point of another vertebra, or vice versa.

Ideally, at this point, the segmentation consisted of all vertebrae visualized in the scan, and no other regions. Quite often though, several vertebrae were connected to each other. Therefore, the final step in improving the binary segmentation was performing a watershed to disconnect any connected vertebrae^[[1]](#footnote-1)^. First, a distance map was created, specifying the smallest distance to the boundary of a vertebra for each voxel. Using this distance map, seeds were determined as regions within vertebrae of which all voxels were at least 2 cm away from the boundary. The final binary segmentation was then created by running the watershed algorithm using the seeds and distance map. Since partially visible vertebrae were often removed by the watershed (because there are no voxels at least 2 cm away from the boundary), all regions smaller than 5 cm^3^ were removed from the segmentation beforehand and reinserted afterwards.

The next step in post-processing was combining the improved binary segmentation with the labeled segmentation to create the final labeled segmentation. First, the identified vertebrae were given a temporary label, ordered consecutively from top to bottom. Then, the regions of the vertebrae in the binary segmentation were inspected in the initial labeled segmentation to determine the frequencies of different labels within each region. The most frequent label within each vertebra was considered to be the predicted label. All labels with a frequency of at least 95% within a vertebra, were considered to be confident predictions. Then, the temporary (consecutively ordered) labels were subtracted from the confidently predicted labels to determine the offset from the temporary labels. Ideally, the confident labels were ordered consecutively and the offset is the same for all labels. The most frequent offset was chosen as the “true” offset and all vertebrae are labeled accordingly, creating the final labeled segmentation. If there were no confidently predicted labels, or if two (or more) offsets were observed equally often, the most confident prediction was considered to be “true”, and other vertebrae were labeled accordingly.

For the combined approach, using only the labeling network, the labeled segmentation was first converted to a binary segmentation and then improved using the same steps as described above. This improved binary segmentation was then combined with the initial labeled segmentation to create the final segmentation, also as described before.

Post-processing was implemented in Python 3.7, using the libraries NumPy 1.19, NiBabel 3.1.1, scikit-image 0.17.2 and SimpleITK 1.2.4.

**Table S3 |** Segmentation results per vertebral level for the internal validation

|  | **Dice similarity coefficient (%)** | | |  | **Hausdorff distance (mm)** | | |  |
| --- | --- | --- | --- | --- | --- | --- | --- | --- |
|  | **Sequential approach** | **Combined approach** | **p** |  | **Sequential approach** | **Combined approach** | **p** |  |
| **T1** | 95.0 (92.3 - 95.9) | 95.8 (94.6 - 96.3) | 0.053 |  | 3.2 (2.8 - 5.3) | 3.6 (2.2 - 4.1) | 0.323 |  |
| **T2** | 95.7 (95.4 - 96.2) | 96.0 (95.2 - 96.6) | 0.229 |  | 3.0 (2.2 - 4.1) | 3.0 (2.2 - 4.4) | 0.670 |  |
| **T3** | 95.8 (94.7 - 96.3) | 96.1 (95.1 - 96.7) | 0.010 |  | 3.3 (2.9 - 4.2) | 3.0 (2.1 - 3.7) | 0.001 |  |
| **T4** | 96.0 (95.2 - 96.5) | 96.3 (95.2 - 96.8) | 0.061 |  | 3.2 (2.4 - 4.4) | 3.0 (2.2 - 4.3) | 0.657 |  |
| **T5** | 95.7 (95.0 - 96.6) | 95.8 (94.3 - 96.9) | 0.404 |  | 3.6 (3.0 - 5.0) | 4.2 (2.8 - 5.2) | 0.559 |  |
| **T6** | 96.3 (94.5 - 96.9) | 96.2 (94.7 - 97.1) | 0.369 |  | 3.2 (2.4 - 5.2) | 4.0 (2.6 - 5.9) | 0.465 |  |
| **T7** | 96.1 (94.4 - 96.9) | 96.0 (94.6 - 97.0) | 0.532 |  | 3.8 (2.9 - 5.1) | 4.2 (2.7 - 5.8) | 0.585 |  |
| **T8** | 96.6 (95.3 - 97.1) | 96.3 (94.6 - 97.1) | 0.849 |  | 3.7 (3.1 - 5.3) | 4.6 (3.0 - 6.4) | 0.509 |  |
| **T9** | 96.5 (94.8 - 97.1) | 96.5 (95.3 - 97.0) | 0.944 |  | 4.9 (3.0 - 6.2) | 4.6 (3.0 - 6.1) | 0.787 |  |
| **T10** | 96.3 (94.9 - 97.1) | 96.3 (95.2 - 97.0) | 0.335 |  | 5.0 (2.9 - 8.0) | 5.2 (3.0 - 7.7) | 0.720 |  |
| **T11** | 96.7 (95.9 - 97.0) | 96.7 (95.9 - 97.1) | 1.000 |  | 4.1 (3.6 - 6.4) | 4.5 (3.2 - 6.3) | 0.670 |  |
| **T12** | 97.0 (95.9 - 97.3) | 97.0 (95.7 - 97.3) | 0.739 |  | 4.7 (3.3 - 6.6) | 4.9 (3.2 - 7.0) | 0.396 |  |
| **L1** | 97.6 (97.4 - 97.8) | 97.6 (97.5 - 97.9) | 0.346 |  | 3.0 (2.8 - 3.7) | 3.0 (2.2 - 4.1) | 0.863 |  |
| **L2** | 97.8 (97.5 - 97.9) | 97.7 (97.4 - 98.0) | 0.032 |  | 3.0 (2.4 - 3.6) | 3.0 (2.2 - 4.0) | 0.607 |  |
| **L3** | 97.6 (97.3 - 97.9) | 97.6 (97.1 - 97.9) | 0.153 |  | 3.0 (2.0 - 4.0) | 3.0 (2.0 - 4.0) | 0.313 |  |
| **L4** | 97.7 (97.5 - 98.0) | 97.8 (97.3 - 98.0) | 0.141 |  | 3.0 (2.1 - 3.9) | 2.8 (2.2 - 4.1) | 0.456 |  |
| **L5** | 96.8 (94.8 - 97.4) | 96.6 (94.7 - 97.3) | 0.609 |  | 4.6 (3.5 - 8.0) | 4.2 (3.0 - 7.1) | 0.611 |  |
| **Thoracic** | 96.1 (95.0 - 96.9) | 96.2 (95.0 - 97.0) | 0.003 |  | 3.7 (2.8 - 5.4) | 4.1 (2.8 - 5.8) | 0.446 |  |
| **Lumbar** | 97.6 (97.3 - 97.9) | 97.6 (97.1 - 97.9) | 0.004 |  | 3.2 (2.4 - 4.0) | 3.0 (2.2 - 4.2) | 0.718 |  |
| **All** | 96.7 (95.5 - 97.4) | 96.7 (95.4 - 97.4) | 0.134 |  | 3.6 (2.8 - 5.1) | 3.6 (2.4 - 5.7) | 0.659 |  |

**Table S4 |** Segmentation results per vertebral level for the external validation

|  | **Dice similarity coefficient (%)** | | |  | **Hausdorff distance (mm)** | | |  |
| --- | --- | --- | --- | --- | --- | --- | --- | --- |
|  | **Sequential approach** | **Combined approach** | **p** |  | **Sequential approach** | **Combined approach** | **p** |  |
| **T1** | 86.1 (79.8 - 92.6) | 94.5 (94.4 - 94.6) | 0.371 |  | 4.9 (4.6 - 5.5) | 23.7 (2.4 - 49.1) | 0.371 |  |
| **T2** | 91.4 (88.4 - 91.8) | 92.5 (86.0 - 93.7) | 0.529 |  | 5.0 (3.5 - 6.4) | 5.6 (2.9 - 26.7) | 0.183 |  |
| **T3** | 92.0 (90.3 - 93.3) | 94.0 (93.2 - 94.5) | 0.402 |  | 4.1 (3.6 - 4.5) | 4.8 (3.2 - 15.6) | 0.294 |  |
| **T4** | 91.7 (90.6 - 93.1) | 93.8 (88.9 - 94.6) | 0.933 |  | 4.5 (3.2 - 5.5) | 4.1 (2.2 - 41.6) | 0.407 |  |
| **T5** | 92.5 (91.4 - 93.5) | 92.4 (88.3 - 94.8) | 0.294 |  | 4.6 (4.5 - 6.2) | 8.1 (3.6 - 25.1) | 0.141 |  |
| **T6** | 92.3 (89.7 - 93.4) | 94.9 (88.7 - 95.1) | 0.800 |  | 5.7 (3.7 - 5.7) | 5.5 (3.5 - 11.8) | 0.447 |  |
| **T7** | 93.0 (92.8 - 93.8) | 92.7 (91.1 - 94.1) | 0.529 |  | 4.1 (3.7 - 5.3) | 7.0 (4.4 - 13.3) | 0.059 |  |
| **T8** | 93.5 (92.7 - 94.1) | 94.0 (93.6 - 94.5) | 0.447 |  | 4.5 (4.0 - 5.1) | 6.0 (4.9 - 12.6) | 0.800 |  |
| **T9** | 94.5 (92.1 - 94.8) | 94.6 (93.5 - 95.2) | 0.813 |  | 4.6 (3.7 - 6.5) | 5.4 (4.1 - 17.5) | 0.011 |  |
| **T10** | 95.2 (94.4 - 95.7) | 95.0 (91.5 - 95.5) | 0.343 |  | 4.5 (3.4 - 7.4) | 9.3 (3.6 - 34.8) | 0.041 |  |
| **T11** | 94.7 (93.1 - 95.8) | 93.5 (87.4 - 95.7) | 0.724 |  | 4.7 (3.3 - 5.4) | 9.1 (3.5 - 10.8) | 0.456 |  |
| **T12** | 95.1 (93.6 - 95.7) | 92.9 (78.7 - 95.1) | 0.014 |  | 4.6 (3.6 - 6.9) | 8.5 (4.3 - 11.0) | 0.069 |  |
| **L1** | 95.6 (95.1 - 96.2) | 95.3 (94.6 - 95.9) | 0.221 |  | 3.6 (3.0 - 4.6) | 5.0 (3.0 - 11.2) | 0.182 |  |
| **L2** | 96.2 (95.4 - 96.7) | 95.7 (93.2 - 96.5) | 0.017 |  | 3.4 (2.8 - 5.9) | 7.7 (4.2 - 37.9) | 0.005 |  |
| **L3** | 96.2 (96.0 - 96.5) | 95.6 (93.9 - 96.3) | 0.021 |  | 4.0 (3.3 - 4.5) | 8.0 (3.6 - 12.3) | 0.013 |  |
| **L4** | 96.2 (95.8 - 96.4) | 95.7 (93.9 - 96.3) | 0.031 |  | 4.1 (3.7 - 5.6) | 7.8 (4.1 - 12.0) | 0.005 |  |
| **L5** | 95.2 (92.3 - 95.8) | 93.0 (91.6 - 95.5) | 0.041 |  | 5.8 (4.6 - 7.8) | 7.5 (5.3 - 11.2) | 0.018 |  |
| **Thoracic** | 93.4 (90.7 - 94.8) | 93.9 (88.9 - 95.1) | 0.093 |  | 4.6 (3.6 - 5.9) | 7.1 (3.6 - 21.3) | < 0.001 |  |
| **Lumbar** | 96.0 (95.0 - 96.4) | 95.4 (92.8 - 96.1) | < 0.001 |  | 4.0 (3.2 - 6.1) | 7.2 (4.0 - 12.4) | < 0.001 |  |
| **All** | 94.5 (91.8 - 95.8) | 94.4 (91.4 - 95.5) | < 0.001 |  | 4.5 (3.4 - 6.0) | 7.1 (3.7 - 15.1) | < 0.001 |  |

1. as adapted from: <http://insightsoftwareconsortium.github.io/SimpleITK-Notebooks/Python_html/35_Segmentation_Shape_Analysis.html> [↑](#footnote-ref-1)
